# Persistent Ascites Revealing Mendelian Susceptibility to Mycobacterial Disease Due to IL‐12Rβ1 Deficiency: A Case Report and Review of the Literature

**DOI:** 10.1155/crii/3587297

**Published:** 2026-08-25

**Authors:** Mohammadreza Dolikhani, Ghazaleh Shakibamaram, Parisa Rashtian, Mohammad Saberi, Azadeh Reshadmanesh, Hosseinali Ghafaripour, Elham Moradian, Seyed Alireza Mahdaviani, Jacinta Bustamante

**Affiliations:** ^1^ School of Medicine, Shahid Beheshti University of Medical Sciences, Tehran, Iran, sbmu.ac.ir; ^2^ Department of Pediatric Gastroenterology, Children’s Medical Center, Tehran University of Medical Sciences, Tehran, Iran, tums.ac.ir; ^3^ Department of Medical Genetics, School of Medicine, Tehran University of Medical Sciences, Tehran, Iran, tums.ac.ir; ^4^ Department of Medical Biotechnology, Faculty of Medical Sciences, Tarbiat Modares University, Tehran, Iran, modares.ac.ir; ^5^ Pediatric Respiratory Disease Research Center, National Research Institute of Tuberculosis and Lung Diseases (NRITLD), Shahid Beheshti University of Medical Sciences, Tehran, Iran, sbmu.ac.ir; ^6^ Laboratory of Human Genetics of Infectious Diseases, University of Texas Southwestern Medical Center, Dallas, Texas, USA, utsouthwestern.edu; ^7^ Center for the Genetics of Host Defense, University of Texas Southwestern Medical Center, Dallas, Texas, USA, utsouthwestern.edu; ^8^ Université Paris Cité, Institut Imagine, Laboratory of Human Genetics of Infectious Diseases, Necker Branch, INSERM UMR 1163, Paris, France, u-paris.fr; ^9^ St. Giles Laboratory of Human Genetics of Infectious Diseases, The Rockefeller University, Rockefeller Branch, New York, USA, rockefeller.edu; ^10^ Study Center for Primary Immunodeficiencies, Assistance Publique-Hôopitaux de Paris (AP-HP), Necker Hospital for Sick Children, Paris, France

**Keywords:** ascites, IL-12Rβ1 deficiency, inborn errors of immunity, interferon-gamma, MSMD

## Abstract

**Background:**

Mendelian susceptibility to mycobacterial disease (MSMD) is a subset of inborn errors of immunity (IEIs) predisposing to clinical infections from less virulent mycobacterial species. Variants in 22 genes, particularly in IL‐12Rβ1, have been identified that impair interferon‐gamma (IFN‐γ)‐mediated immunity. We report a new rare MSMD patient with a homozygous *IL12RB1* variant who presented with severe and persistent ascites.

**Case Report:**

We investigated a 30‐month‐old girl who developed an abscess and lymphadenopathy (LAP) in the left axilla after receiving a BCG vaccine. She was diagnosed and treated for BCG‐osis. She presented with ascites, which gradually worsened, along with symptoms of liver dysfunction. Her portal hypertension (PHT) was considered the most likely cause of ascites after exclusion of other common causes. The whole exome sequencing (WES) revealed a homozygous rare variant (c.635G>A; p.Arg212Gln) in the *IL12RB1* gene. Sanger sequencing analysis confirmed that both parents were heterozygous carriers of the variant. She made a good recovery after receiving the appropriate antimycobacterial and ascites treatments, with significant resolution of her symptoms. Because disseminated *M. bovis* BCG infection occurred in the setting of IL‐12Rβ1 deficiency, prolonged susceptibility‐guided antimycobacterial therapy with adjunctive IFN‐γ1b and close follow‐up by infectious disease and immunology, was planned.

**Conclusion:**

This case highlights severe persistent ascites as an unusual manifestation of PHT in IL‐12Rβ1 deficiency associated with MSMD. Evaluation for PHT should be considered in MSMD patients with abdominal distension, gastrointestinal symptoms, hepatosplenomegaly, abnormal liver function tests, growth failure, or unexplained free abdominal fluid.

## 1. Introduction

Primary immunodeficiencies (PIDs), now more commonly referred to as inborn errors of immunity (IEIs), constitute a heterogeneous group of disorders caused by defects in immune development, regulation, or function [1]. Mendelian susceptibility to mycobacterial disease (MSMD) is a group of IEI disorders in which defects in the intrinsic and innate immune systems predispose patients to infection with less virulent *Mycobacterium* species, such as *Mycobacterium bovis* (BCG vaccine strain) and environmental mycobacteria (EM). More virulent organisms, such as *Mycobacterium tuberculosis*, as well as intracellular pathogens, such as *Candida* spp. and *Salmonella* spp., have also been reported [2, 3]. MSMD patients present clinically with BCG‐osis (disseminated BCG infection), BCG‐itis (regional or localized infection after BCG vaccination), growth retardation, hepatomegaly, mycobacterial lung disease, leukocytoclastic vasculitis, and osteomyelitis [4–6]. To date, mutations in multiple genes have been identified, including *CYBB*, *CCR2*, *IKBKG*, *IL12B*, *IL12RB1*, *IL12RB2*, *IL23R*, *IFNG*, *IFNGR1*, *IFNGR2*, *IRF1*, *IRF8*, *ISG15*, *JAK1*, *MCTS1*, *RORC*, *SPPL2A*, *STAT1*, *TYK2*, *USP18* and *ZNFX1* [7–12]; in signaling IL‐12, IL‐23, or ISG15, and in the effective production of interferon‐gamma (IFN‐γ) have been identified [3, 6, 13, 14].

Under the Iranian national vaccination programme, newborns must receive the live attenuated BCG vaccine (Pasteur 1173P2) [15–17]. Describing new clinical manifestations of MSMD caused by defects in IFN‐γ–mediated immunity refines the disease spectrum and clarifies the underlying pathogenesis. These insights enable earlier recognition and inform the development of targeted, evidence‐based treatment strategies. This case report describes a 3‐year‐old girl who presented with severe, persistent ascites due to MSMD with a rare variant of *IL12RB1*. Very few cases of ascites have been reported in the literature as a complication of MSMD, particularly in IL‐12Rβ1 deficiency [13, 18–20].

Ascites in patients with MSMD may arise through different mechanisms, including infectious peritonitis or portal hypertension (PHT), which is related to hepatic or lymphatic involvement. Given the significant consequences for growth, it is imperative to outline care and treatment for this medical condition, given the lack of specific guidelines and the rarity of the case.

## 2. Case Report

### 2.1. Age 3 Months: BCG Vaccination

The patient is an Iranian girl, apparently born to nonconsanguineous parents. According to her vaccination record, she received the BCG vaccine at 3 months of age.

### 2.2. Age 5 Months: First Admission

Two months later, she was hospitalized with symptoms of BCG‐itis, including an abscess and lymphadenopathy (LAP) in the left axilla and on the left side of the neck. The patient underwent surgery to remove the affected lymph nodes and drain the abscess. Pathology revealed necrotizing granulomatous lymphadenitis with numerous acid‐fast bacilli (AFB). She was then treated with ethambutol (20 mg/kg), rifampin (10 mg/kg), isoniazid (15 mg/kg), ciprofloxacin (20 mg/kg), and clarithromycin (15 mg/kg) for a 12‐month regimen.

**Figure 1:**
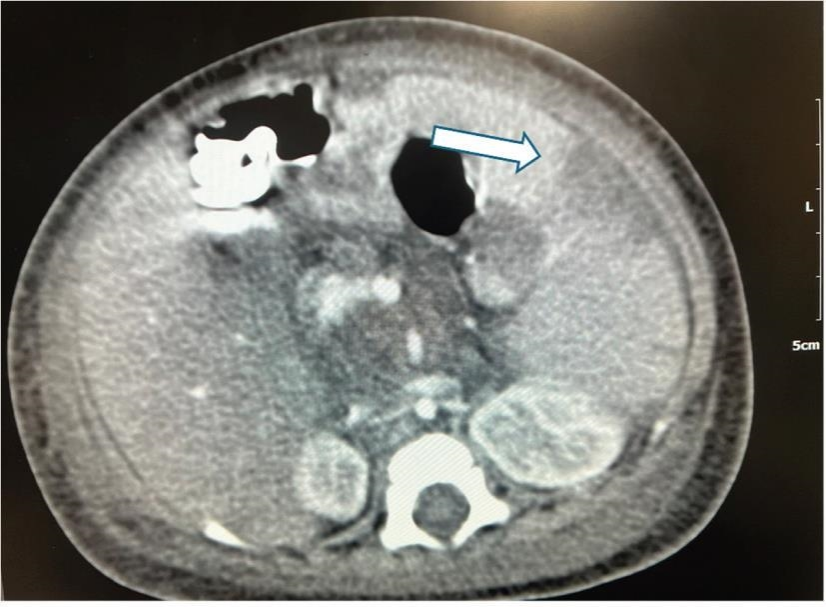
Hypodense wedge‐shaped splenic lesion on abdominal computed tomography.

Sonography indicated pericardial effusion, splenomegaly (98 mm), and mild free abdominal fluid. The computed tomography (CT) scan with and without contrast showed a hypodense, wedge‐shaped area in the lower aspect of the spleen, as shown in Figure 1, as well as several LAPs at the porta hepatis. The purified protein derivative (PPD) test was reported to be negative.

### 2.3. Age 10 Months: Second Admission

Five months after her initial hospitalization, the patient was readmitted with fever and diarrhea. Laboratory evaluation showed elevated liver enzymes, including AST and ALT, as detailed in Table 1. During this admission, she was diagnosed with SARS‐CoV‐2 infection based on a positive PCR test and received supportive care only; no COVID‐19‐specific antiviral or immunomodulatory therapy was administered. No organism was isolated from buffy coat or gastric washing AFB studies. Microbiological investigation, including two AFB examinations of buffy coat and gastric washing samples, yielded negative results. Radiologic assessment with abdominal and pelvic CT revealed a mild left‐sided pleural effusion with adjacent subsegmental collapse‐consolidation, as well as mild free fluid within the pelvic cavity. The stool examination (S/E) confirmed *Candida* spp. infection. Antimycobacterial therapy was temporarily discontinued because of liver enzyme elevation and was reintroduced at discharge after improvement of liver function tests and reassessment by the infectious disease team. Because of severe anemia, with a hemoglobin level of 5.6 g/dL, she received three units of packed red blood cells. Endoscopy showed grade 1 esophageal varices and PHT.

**Table 1:** Serial hematological and immunological laboratory results.

Parameter	First admission	Second admission	The third admission	The final admission	Normal ranges
WBC (×10^3^/µL)	24…7.2	16.6… 14.7	18.7	5.9	5–14.5
Hemoglobin (g/dL)	11 … 7.7	5.6 …7.6…8.6	11.3	12.9	10.5–14.0
Platelets (/μL)	297 … 350	275..440	382	251	150,000–450,000
ESR 1 h (mm/h)	27 … 5	—	—	10	0–20
CRP (mg/L)	32	—	—	19	<0.1
AST (U/L)	70	210…86	—	45…285…41	6–45
ALT (U/L)	18	16…25	—	24..156…29	6–45
ALP (U/L)	165	210	—	434..454…656	91–334
Albumin (g/dL)	2.6	—	—	—	3.5–5.5
PT (INR) (s)	16.2 (1.2)	—	38.2 (3.75) …. 13.5 (1)	12.8 (1.07)	12.2–15.5
PTT (s)	33	—	29	28	26.5–35.5
IgG (mg/dL)	—	830	—	462	442–1139
IgE (mIU/mL)	—	4	—	6	Up to 68
IgM (mg/dL)	—	61	—	44	43–184
IgA (mg/dL)	—	55	—	—	21–150

Flow cytometric analysis of lymphocyte subsets was performed, as detailed in Table 2.

**Table 2:** Flow cytometric analysis of lymphocyte subsets.

Lymphocyte subsets	Blood levels	Reference range
CD3	43	65%–85%
CD4	27	40%–60%
CD8	14	20%–40%
CD4/CD8	1.92	1.5–2.5
CD19	29	14–33
CD20	35	15.7–35.6
NK cells	21.2	4–19
Memory CD4	8	9–26
Memory CD8	11	4–16
Naïve CD4	84	53–86
Naïve CD8	81	69–97

### 2.4. Interval Course Between Admissions

Between admissions, the patient remained symptomatic, with intermittent abdominal distension, recurrent diarrhea, poor oral intake, failure to thrive, and poor weight gain. These symptoms gradually worsened, culminating in recurrent admissions for ascites, gastrointestinal symptoms, and liver dysfunction.

Whole exome sequencing (WES) was performed using the Twist Custom Target Enrichment kit (Twist Bioscience, San Francisco, CA, USA) on the NovaSeq platform (Illumina, Inc., San Diego, CA, USA), with a mean sequencing depth of 272x. A homozygous missense variant mutation (c.635G>A; p.Arg212Gln) in exon 7 of the *IL12RB1* (NM_005535.3) gene was identified. It was predicted to be deleterious by PolyPhen‐2 (0.99) and CADD (21.5) pathogenicity prediction tools. Population data analysis revealed a very low allele frequency of 0.0004%, with no documented instances of homozygosity in the Genome Aggregation Database v 4.1 (gnomAD). Arginine, a positively charged residue, is replaced by glutamine, which carries a neutral polar side chain, potentially altering protein folding, stability, or function, leading to a deficiency of IL‐12Rß1. This variant has been documented in the HGMD and ClinVar databases as disease‐causing [21, 22]. The patient’s parents were tested, and both parents were confirmed to be heterozygous carriers of the mentioned variant. Notably, their older daughter, who was 2 years older than the affected patient, was clinically healthy despite receiving vaccinations; however, genetic testing was not performed for her. Despite the absence of a reported consanguineous relationship, the parents belong to the same extended family, which could account for the patient’s homozygosity.

### 2.5. Age 19 Months: Third Admission

Nine months later, the patient was readmitted with persistent abdominal pain, vomiting, poor weight gain, and ongoing diarrhea. Grade 1 esophageal varices, PHT, mild ascites, splenomegaly (98 mm), necrotic para‐aortic lymph nodes, and increased liver echogenicity were observed. The patient also developed epistaxis, hematoma, and coagulopathy, with a prothrombin time (PT) of 38.2 s and an international normalized ratio (INR) of 3.75, in association with abnormal liver tests. No definite organism was isolated during this admission. The hepatic dysfunction was considered multifactorial, possibly related to PHT physiology, chronic inflammatory involvement, and disseminated BCG‐related diseases. She was treated with fresh frozen plasma (FFP), intravenous albumin, furosemide, and therapeutic paracentesis. The combination of recurrent ascites, splenomegaly, esophageal varices, and liver dysfunction suggested progressive hepatobiliary involvement with portal‐hypertensive physiology.

### 2.6. Age 30 Months: Final Admission

**Figure 2:**
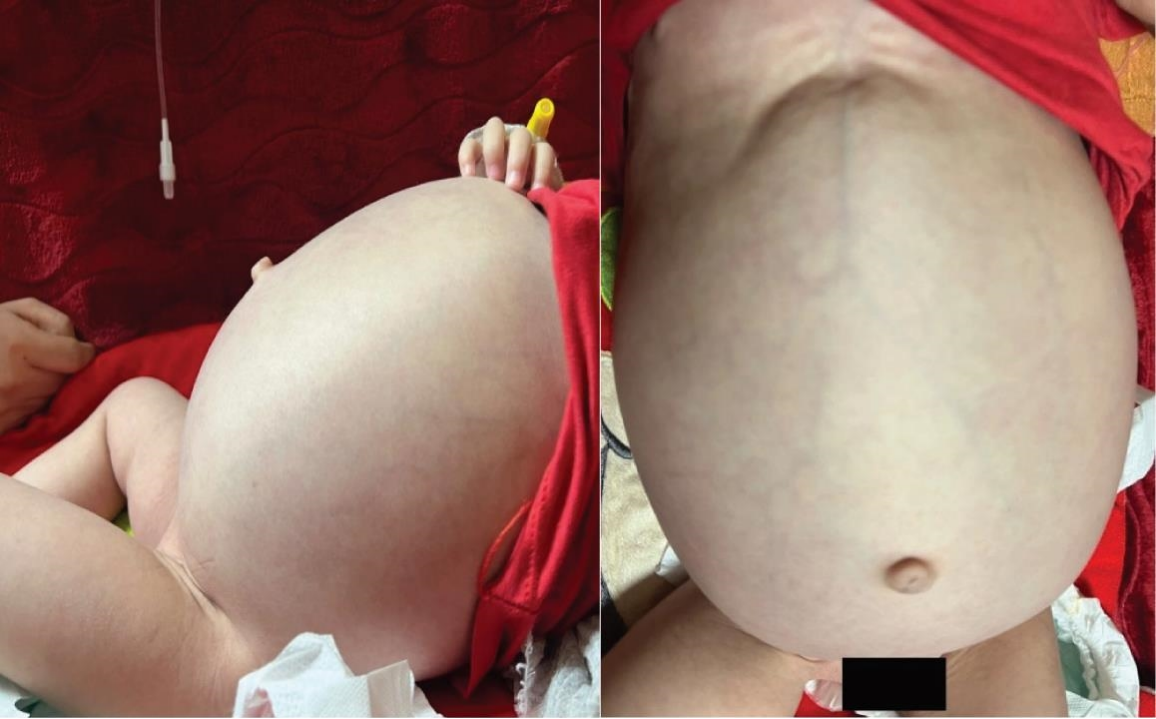
The patient’s abdominal ascites during the last hospitalization.

**Figure 3:**
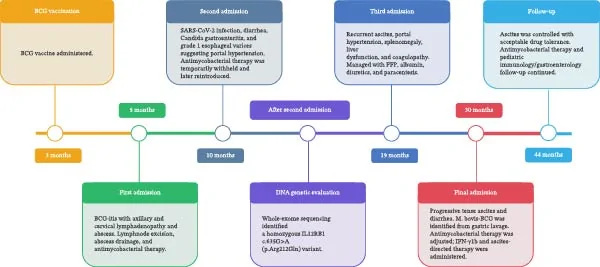
Timeline of admissions and treatments.

The patient was referred to Masih Daneshvari Hospital due to progressive ascites and diarrhea. Her weight was 9 kg and her height was 77 cm (body mass index: 15.2 kg/m^2^), indicating growth failure (CDC Z‐score: −1). Physical examination revealed stable vital signs and marked abdominal distension with tense ascites (Figure 2). Ascitic fluid analysis showed a transudate with a high serum‐ascites albumin gradient (SAAG). Direct smear, culture, and PCR tests were negative for bacterial peritonitis. Serological tests for HCV, HBV, HIV, EBV, and CMV were negative, as was fecal culture. Chest radiography was unremarkable. Gastric lavage identified *M. bovis* BCG as sensitive to rifampin and ethambutol. A detailed timeline of her hospitalization and treatment is shown in Figure 3.

### 2.7. Treatment and Follow‐Up

She was treated with levofloxacin (7–15 mg/kg), amikacin (15 mg/kg), isoniazid (5–10 mg/kg), and ethambutol (15 mg/kg). Rifaximin (10 mg/kg) was added as intestinal decontamination therapy in the setting of severe PHT, recurrent ascites, and gastrointestinal symptoms. Spironolactone (2 mg/kg), albumin, and furosemide were used to control ascites. Subcutaneous IFN‐γ1b was administered at 20 µg every other day as adjunctive immunotherapy alongside susceptibility‐guided antimycobacterial therapy for two doses. Alpha‐1 antitrypsin levels and the insulin tolerance test were normal. After marked clinical improvement, reduction in ascites, and improved liver function, she was discharged on spironolactone (5 mg/kg/day), furosemide (2 mg/kg/day), isoniazid (10 mg/kg/day), ethambutol (15 mg/kg/day), levofloxacin (7 mg/kg/day), and amikacin (20 mg/kg/day).

### 2.8. Age 44 Months: Follow‐Up

At the latest documented follow‐up, at 44 months of age, ascites was controlled, and drug tolerance was acceptable. Subsequent shear wave elastography showed a liver size of 9.4 cm, a mean elasticity of 5.9 kPa, and a fat fraction of 3%, ruling out a fatty liver or significant fibrosis. The recurrent ascites was therefore attributed to portal‐hypertensive physiology, most likely related to chronic inflammatory hepatobiliary or porta‐hepatis involvement in the setting of BCG‐osis; however, no definitive portal venous anomaly was documented. The planned long‐term management included prolonged susceptibility‐guided antimycobacterial therapy, with treatment duration to be individualized according to the clinical response, microbiological findings, drug tolerance, and multidisciplinary follow‐up.

## 3. Discussion

This case report describes a patient with IL‐12Rβ1 deficiency who developed *M. bovis* BCG vaccine‐strain infection and subsequently developed treatment‐resistant ascites related to PHT. The most common manifestation of BCG‐osis is LAP, which was the first sign in this case [6, 23]. Histopathological examination showed AFB, leading to a diagnosis of BCG‐osis, which was treated with second‐line antimycobacterial drug regimens [24]. Whole‐exome sequencing (WES) identified a homozygous likely pathogenic variant in *IL12RB1* (c.635G>A; p.Arg212Gln), substituting a conserved arginine with glutamine within the receptor’s extracellular cytokine‐binding domain, specifically the fibronectin type III domain.

IL‐12Rβ1 is a shared receptor subunit for IL‐12 and IL‐23 and plays a central role in antimycobacterial immunity [3, 9, 25]. IL‐12Rβ1 deficiency impairs IL‐12/IL‐23–dependent IFN‐γ production by T cells and natural killer cells [3, 14, 25]. Reduced IFN‐γ production limits macrophage activation and compromises the intracellular killing of phagocytosed mycobacteria [3, 25]. This defect explains the susceptibility of affected patients to BCG‐osis, BCG‐itis, environmental mycobacterial infections, and other infections by intramacrophage pathogens [3, 6, 14, 25]. The p.Arg212Gln variant identified in our patient has previously been shown to impair IL‐12Rβ1 membrane localization and to disrupt IL‐12/IL‐23–IFN‐γ signaling [22, 25]. This mechanism plausibly explains the patient’s susceptibility to disseminated BCG and Candida infections.

The c.635G>A variant has previously been reported in MSMD patients with BCG‐osis and candidiasis, but its association with ascites indicates a novel phenotypic expansion. Previous reports of IL‐12Rβ1 deficiency have described gastrointestinal or abdominal involvement, including enteropathy, hypogammaglobulinaemia, diarrhea, hepatosplenomegaly, lymphadenitis, and ascites [13, 18, 19]. The c.635G>A *IL12RB1* variant has also been reported in patients with MSMD who presented with BCG‐osis and candidiasis [22]. However, to our knowledge, the association of this variant with severe PHT‐related ascites has not been reported, suggesting a possible expansion of the clinical phenotype.

In our patient, recurrent and persistent ascites were accompanied by findings suggestive of PHT, including splenomegaly, esophageal varices, abnormal liver function tests, and high‐SAAG transudative ascitic fluid [26, 27]. In IL‐12Rβ1 deficiency, impaired IL‐12/IL‐23–IFN‐γ signaling compromises macrophage activation and host defense against mycobacterial infections, potentially allowing persistent or disseminated infection [3, 25]. Therefore, we hypothesize that chronic mycobacterial‐driven inflammation, possibly together with porta‐hepatis LAP or lymphatic involvement, may have contributed to hepatic or portal vascular dysfunction in this patient. However, because the exact mechanism was not directly proven, PHT–related ascites should be considered a plausible complication in this case rather than a confirmed direct consequence of the IL12RB1 variant.

Interestingly, the same missense variant c.635G>A (p.Arg212Gln) in the *IL12RB1* gene has previously been reported in two unrelated patients with MSMD. Zhou et al. [22] described a Chinese patient with compound heterozygous mutations (p.Arg212Gln and c.765delG) who developed localized BCG‐osis but did not present with ascites or liver complications. Similarly, Rosain et al. [21] identified this variant in an Iranian patient as part of a compound heterozygous genotype (p.Arg212Gln and a duplication of exons 10–12), who exhibited classical MSMD features without gastrointestinal involvement. Functional studies by Zhou et al. [22] confirmed that the p.Arg212Gln substitution disrupts membrane localization of IL‐12Rβ1, resulting in impaired IFN‐γ signaling. However, our patient, homozygous for this variant, developed severe PHT, ascites, and growth impairment, representing a novel and more severe clinical phenotype associated with this mutation. This may suggest a dosage effect or increased pathogenicity when homozygous.

In this patient, the ascitic fluid was transudate. Generally, ascites are classified as portal hypertensive, nonportal, or mixed [28]. PHT ascites result from liver cirrhosis, cardiac failure, hepatic sinusoidal obstruction syndrome, Budd–Chiari syndrome, and portal vein thrombosis [29]. PHT has already been reported as a complication of disseminated histoplasmosis in several cases of MSMD [26]. In this patient, porta‐hepatis LAP and portal‐hypertensive findings suggested that local inflammatory or lymphatic involvement may have contributed to impaired portal venous drainage and ascites formation. However, no definite portal vein anomaly was confirmed, so this proposed mechanism remains speculative.

Arias et al. [18] reported a patient with disseminated *Mycobacterium tuberculosis* infection, severe enteropathy, and hypogammaglobulinaemia. Gastrointestinal inflammation is likely to cause protein loss, leading to ascites [18]. Dizaj et al. [19] reported a patient with ascites linked to systemic infection, suggesting that inflammatory damage and lymphatic congestion in the liver or at the porta hepatis may contribute to ascites. Al‐Kzayer et al. [13] reported a patient with IL‐12Rβ1 deficiency, highlighting hepatosplenomegaly and ascites. In these patients, ascites is likely a secondary complication of PHT due to LAP at the porta hepatis or direct inflammation of the liver parenchyma [13]. Tassone et al. [20] described an adult patient with disseminated *Mycobacterium genavense* infection after immunosuppressive therapy who harbored compound heterozygous mutations in the IL‐12 receptor gene. Although this case involved an older patient and was complicated by immunosuppression, the report highlights the potential for severe gastrointestinal involvement, including chronic diarrhea and ascites, in patients with defects in the IL‐12/IL‐23/IFN‐γ axis. Although all of the mentioned studies reported patients with ascites, none of the previously reported cases described massive, persistent, or refractory ascites comparable to the present case. Table 3 summarizes key clinical, laboratory, and imaging findings associated with gastrointestinal manifestations of IL‐12Rβ1 deficiency, as reported in the literature.

**Table 3:** Review of gastrointestinal manifestations and ascites in IL‐12Rβ1 deficiency cases.

Reference	Patient details and age	Gastrointestinal manifestations	Ascites findings
Arias et al. [18]	2‐year‐old boy with IL‐12Rβ1 deficiency and disseminated tuberculosis	Chronic enteropathy, growth retardation, hypogammaglobulinemia	Severe ascites from portal hypertension, likely due to chronic gastrointestinal inflammation
Dizaj et al. [19]	8‐year‐old girl, 7‐year‐old boy, 3‐year‐old boy; with IL‐12Rβ1 mutations	Lymphadenopathy and signs of mycobacterial infection	One patient showed abnormal liver enzymes, suggesting a potential link to systemic infection
Al‐Kzayer et al. [13]	8‐year‐old girl with IL 12Rβ1 deficiency	Lymphadenopathy following BCG vaccination and hepatosplenomegaly	Ascites with imaging showing portal hypertension and enlarged porta‐hepatis lymph nodes
Tassone et al. [20]	35‐year‐old woman disseminated *Mycobacterium genavense* post‐immunosuppressive therapy	Chronic diarrhea, intestinal lymphangiectasia, and protein‐losing enteropathy	Ascites with confirmed hypoalbuminemia

## 4. Conclusion

This case expands the recognized clinical spectrum of *IL-12Rb1* deficiency by highlighting severe, persistent PHT‐related ascites as a rare manifestation of MSMD. In patients with MSMD, abdominal distension, hepatosplenomegaly, abnormal liver function tests, gastrointestinal symptoms, growth failure, or unexplained free abdominal fluid should prompt evaluation for PHT and hepatobiliary involvement. Early recognition, genetic diagnosis, susceptibility‐guided antimycobacterial therapy, and multidisciplinary follow‐up may improve clinical outcomes in similar patients.

## Author Contributions

Mohammadreza Dolikhani, Ghazaleh Shakibamaram, and Parisa Rashtian contributed to the conception and design of the case report and were involved in drafting the manuscript. Mohammad Saberi and Azadeh Reshadmanesh carried out the genetic analyses and contributed to the interpretation of the molecular data. Hosseinali Ghafaripour and Elham Moradian participated in the patient’s clinical management and provided critical insights into the diagnosis and treatment. Seyed Alireza Mahdaviani supervised the overall study, reviewed, and revised the manuscript critically for important intellectual content. Jacinta Bustamante provided expert opinion on the immunological and genetic findings and substantively revised the final version of the manuscript.

## Funding

The authors received no financial support for the research, authorship, or publication of this article.

## Disclosure

All authors have read and approved the final version of the manuscript and agree to be accountable for all aspects of the work.

## Ethics Statement

According to the Ethics Committee of Shahid Beheshti University of Medical Sciences, ethical approval was not required for this case report, as no experimental intervention or procedure beyond routine clinical care was performed. This study was conducted in accordance with the principles of the Declaration of Helsinki.

## Consent

Written informed consent for participation in the study and for the publication of the case details was obtained from the patient’s parents.

## Conflicts of Interest

The authors declare no conflicts of interest.

## Patient Perspective

The parents said, “We are happy as our baby has improved, thanks to the medical team.” The National Research Institute of Tuberculosis and Lung Diseases (NRITLD) will care for and closely follow‐up with her to ensure the best possible outcomes for this patient and her family.

## Data Availability

The raw sequence data reported in this study have been deposited in the Genome Sequence Archive (Genomics, Proteomics & Bioinformatics 2021) at the National Genomics Data Center (Nucleic Acids Res 2022), China National Center for Bioinformation/Beijing Institute of Genomics, Chinese Academy of Sciences, under GSA‐Human Accession Number HRA011708. The data are publicly accessible at https://ngdc.cncb.ac.cn/gsa-human/ [27, 30].
